# Dr. Sage’s Catarrh Remedy

**Published:** 1874-09

**Authors:** 


DR. SAGE'S CATARRH REMEDY.—A mixture in the following proportions very closely resembles Dr. Sage's Catarrh Remedy:

| | |
|---|---|
| ℞.—Hydrastis canadensis, . . . . . | gr. v. |
| Indigo, . . . . . . . | gr. ss. |
| Camphoræ pulv., | |
| Acidi carbolici, . . . . . | *aa* gr. ij. |
| Sodii chloridi, . . . . . | gr. l. |

Powder the camphor by means of a drop of alcohol, and mix with the salt, previously reduced to a moderately fine powder; rub the indigo and carbolic acid together, mix with the salt and camphor, and lastly add the powdered hydrastis, and mix intimately, without much pressure, in a mortar.

A NOVEL IDEA OF PLEASURE.—The *Boston Journal of Chemistry* says: "It is pleasant to learn that an enormous deposit of Epsom salts has been discovered at Alacandre, in Spain.

